# The Black radical imagination: a space of hope and possible futures

**DOI:** 10.3389/fneur.2023.1241922

**Published:** 2023-09-22

**Authors:** Tanisha G. Hill-Jarrett

**Affiliations:** ^1^Global Brain Health Institute, University of California, San Francisco, San Francisco, CA, United States; ^2^Department of Neurology, Memory and Aging Center, School of Medicine, University of California, San Francisco, San Francisco, CA, United States

**Keywords:** imagination, Afrofuturism, alternative futures, health justice, Black aging, neurocognition, values

## Abstract

The radical imagination entails stepping outside the confines of the *now* and into the expansiveness of *what could be*. It has been described as the ability to dream of possible futures and bring these possibilities back to the present to drive social transformation. This perspective paper seeks to provide an overview of the radical imagination and its intersections with Afrofuturism, a framework and artistic epistemology that expresses the Black cultural experience through a space of hope where Blackness is integral. In this paper, I propose three processes that comprise the radical imagination: (1) imagining alternative Black futures, (2) radical hope, and (3) collective courage. I consider the neural networks that underlie each process and consider how the Black radical imagination is a portal through which aging Black adults experience hope and envision futures that drive social change. I conclude with considerations of what brain health and healing justice looks like for aging Black Americans— specifically, how invocation of the Black radical imagination may have positive brain health effects for a demographic group at increased risk for Alzheimer’s disease and related dementias.

## 1. Introduction

“Colonization of the imagination is the most dangerous and subversive form there is: for it is where all other forms of decolonization are born. When the imagination is unshackled, liberation is limitless.”

-Walidah Imarisha.

Our thoughts create our reality. In a world where racism, sexism, homophobia, ageism and other forms of oppression maintain a false social hierarchy and unequal distribution of power, the radical imagination is the foundation to re-envisioning the future and building a new, balanced world. Defined as the “ability to imagine the world, life and social institutions, not as they are but as they might otherwise be,” the radical imagination brings “possible futures ‘back’ to work on the present, to inspire action” (1). To fully unpack the radical imagination requires consideration of its etymology. While the word “radical” in today’s day and age often has a negative connotation and is used synonymously with extremism, “radical” is derived from the Latin word, *radix*, which translates to “root.” Perhaps not coincidentally, ideas that are deemed “radical” are oftentimes grounded in the understanding and/or identification of systemic inequity, oppression, and power imbalance as root causes of societal problems (2). To be radical, in its truest form, is to get to the root of the problem.

The imagination is the generative foundation for all new ideas and involves the social and cultural creation of meaning (3). Early conceptions of the imagination included the belief that imagining was restricted to solely visual mental imagery (4), but it is now known that the imagination encompasses other perceptual modalities [e.g., auditory, tactile, olfaction; motoric (5, 6)] and underlies prosocial behavior [e.g., empathy (7, 8)] at both the individual and collective levels. As will later be discussed, it is the collective imagination – a group process through which knowledge and new ideas are co-produced (9)– that undergirds many social justice movements toward Black collective liberation. The imagination also exists on a temporal plane in that it can be oriented toward past happenings, alternative presents, or possible futures (3). All three aspects of time are inextricably linked and deeply racialized (10, 11). For example, Black Americans are unjustly cut from accessing all elements of time: (1) they are cut from their past and family lineage via the transatlantic slave trade (11); (2) cut from the present via labor inequities that are rooted in capitalism and the historical commodification of the Black body for capital, which impact access to time, rest, and leisure (12–14) and (3) cut from the future via premature deaths and abbreviated lifespans evidenced by health disparities (15–20), mass incarceration (21, 22), and state-sanctioned violence (23, 24). It is through this intentional manipulation and monopolizing of time that the imagination is squelched, and social and systemic inequities persist. Thus, for Black Americans, the collective invocation of the imagination is a longstanding form of resistance and a critical means of making it to the future. In a society where aging is a privilege, the Black imagination is a tool for survival.

“Any serious motion toward freedom must begin in the mind.” (25) The imagination is a mechanism through which we are not only able to envision new, freer worlds, but we can actualize them. To *radically* reimagine requires more than the deconstruction of problematic policies or dismantling existing power structures (although both are important components of it); it additionally encompasses the process of *rebuilding* from a space of possibility. The radical imagination extends beyond mere fantasy. It is a central driver of cognition and perception in that society can only create that which its members can imagine (9, 26). When equipped with deep understanding and acknowledgment of the past, the radical imagination can become a portal to a more just, equitable word (27).

In this perspective paper, I argue there are three components of the Black radical imagination which include: (1) imagining alternative Black futures, (2) radical hope, and (3) collective courage. I explore the neuroanatomical underpinnings of these processes and values, and then consider their relationship to brain health. Lastly, I delineate how the Black radical imagination is a necessary conduit to possible futures rooted in health and healing justice for Black Americans. I reflect on what it means to disrupt existing power imbalances and place Black Americans as the central story writers and designers of an equitable future.

## 2. Imagining alternative Black futures

People of the African diaspora have long been building new futures to escape trauma and cultivate shared realities. The paradox and illegitimacy of ancient Western criticism was that people from the continent of Africa were considered lacking in imagination and incapable of “true sophistication” required for social evolution, while also, ironically, deemed “too imaginative” in their belief of the supernatural, deities, Dogon myths, and orishas (28). Dating back as far as 500–300 BCE (29), these traditional ideas and belief systems within African societies were the earliest forms of Black imagination and ways of existing that countered the Western gaze.

The term “Black radical imagination,” first appearing in Robin D.G. Kelly’s, Freedom’s Dreams (25), is the process of Black people collectively envisioning a revolutionary and liberated future. Early conjuring of the Black radical imagination is apparent in the kidnapping and forced relocation of ~12.5 million people from the continent of Africa to the Americas via the transatlantic slave trade from 1500 to 1865. This treacherous and inhumane journey overseas required those captured to imagine alternative forms of resistance that ranged from refusing to eat to staged ship revolts. Some even made the difficult decision to jump overboard to cheat the ultimate death: enslavement and control.

Upon arrival to the Americas, through collective struggle and solidarity, those who were Black and enslaved identified ways to undermine slavery and actualize their birthright: freedom. Despite attempts to erase the enslaved of their culture and customs, commodify their labor, and indoctrinate them into Western practices, Black people resisted through subtle acts of defiance (e.g., sabotaging crops, damaging equipment, feigning illness) and through clever communication and escape tactics in this foreign land. For example, the different hairstyles Black women wore contained intricate designs like conrows or Bantu knots and were used as maps hidden in plain sight to escape to freedom (30). Women also hid rice and seeds in their hair to grow once they escaped to a free territory. Songs contained embedded messages for those escaping North to freedom via the secret routes of the Underground Railroad. The spiritual, “Wade in the Water,” was sung by enslaved persons to warn those enroute to freedom to avoid trails and use the river to hide their body scent and to evade search dogs (31). Harriet Tubman, an abolitionist and former slave who escaped to freedom, is one of the most widely known conductors of the Underground Railroad. It was her ability to dream of new worlds and incorporate celestial knowledge (using the North Star as a guide) that allowed her to make 13 rescue missions to help others achieve freedom in the North. Although lesser known, Tubman was also a spy in the Union Army and the leader behind the Combahee River raid, freeing 800 enslaved people (32, 33). It was these visions of alternative (free) futures, resourcefulness, and ingenious strategizing that have long carried Black Americans through turbulent times.

The Black radical imagination has been a cornerstone of Black American culture and the social struggle for equity that exists to this day. This collective dreaming of alternative futures is the impetus behind U.S. movements and initiatives for liberation (e.g., Civil Rights Movement, Freedom Riders, March on Washington, Black Lives Matter). Imagining in this context is not a discrete process, but one of co-creation that shapeshifts over time and with the needs of the marginalized community (1). It is shaped by the material and social conditions of society (34). The Black imagination remains a site of resistance to oppression to this day. The process of imagining as a liberation praxis remains integral to the generation of Black possible futures (35).

### 2.1. Afrofuturism and the imagination

“The Black voice is forced to be imaginative because otherwise it will be silenced.”

-Jonathan Horstmann

Afrofuturism intersects with the Black imagination to provide a framework for communicating ideas around possible futures and creating spaces of Black empowerment. Afrofuturism is an epistemology and form of artistic expression that explores the African diaspora experience through alternate realities and futures using imagination, technology, and mysticism (36). Coined in 1993 by Mark Dery in his essay “Black to the Future” (37), the practice of Afrofuturism dates to the narrative work of the enslaved and abolitionist discourse of the 18th and 19th centuries (28, 38). Afrofuturism reasserts Black agency in a way that places people of the African diaspora as central storywriters and occupants of past, present, and future. Importantly, the Afrofuturist is not ignorant of historical happenings, but they are not limited by it either (39).

In fact, Afrofuturism has been used by Black American artists, activists, scholars and others as a framework to reclaim and unveil lost histories (40) and reimagine the future through a lens of hope where Blackness is integral and all Black people are safe. It directly counters society’s failure to “articulate and witness Black life” free of struggle (41). The creation of counternarratives and counter futures that upend stereotypes and give Black people control over their image is core to Afrofuturism. Thus, Afrofuturists determine *who* is seen and *how* they are seen. From Octavia Butler’s pioneering science fiction and speculative writings to Sun Ra and the Arkestra’s jazz compilations to the visuals and narrative of the movie Black Panther, Afrofuturism, spans mediums and leans fully into the expansiveness of Black possibility. Existing research denotes the benefit of Black people envisioning possible futures in which they are central (42). Afrofuturism has been utilized as a “praxis for designing equitable futures centered around joy and healing” (43). Because Black bodies are politicized in society, Afrofuturism is political, transformative, and revolutionary (44).

### 2.2. Cognitive neuroscience of the imagination

A cognitive neuroscience perspective considers imagination to be the representational engagement with that which is absent. Abraham (6) synthesized empirical data and theory from cognitive neuroscience literature which resulted in five ways to categorize the imagination within a single framework: mental imagery (perceptual/motor) imagination, intentionality imagination, novel combinatorial imagination, phenomenology imagination, and altered states of imagination. While these processes are described as distinct, they are not independent of one another.

Mental imagery-based imagination is the representation of a sensory experience without direct external stimulus (45). There is evidence that brain regions responsible for sensory perception or motor generation are also activated when those processes are imagined (45). For instance, imagining the sounds of a song activates the auditory cortex (46) and imagining hand movement activates the premotor cortex (47) which maintains the same somatotopic organization in mental representation (48).

Intentionality imagination encompasses both the recollective and social domains and requires one to make appraisals or some form of judgment about actions or events (that could be geared toward the past or the future). Abraham (6) indicated that this could be imagination involving autobiographical or episodic memory (e.g., remembering high school prom), theory of mind (e.g., considering what another person is thinking), self-referential thinking (e.g., considering your own thoughts), or moral reasoning (e.g., evaluating the acceptability of another’s actions). These elements of imagination contain a shared functional neuroanatomical architecture of the default mode network. Specifically, the default mode network consists of the medial prefrontal cortex, retrosplenial and posterior cingulate cortices, anterior lateral temporal cortex, inferior parietal cortex, and mesial temporal lobe (6, 49).

Novel combinatorial imagination consists of the generation of new ideas via creativity, openness, and consideration of the unknown. Abraham (6) noted that key elements of this form of imagination involves extending beyond the status quo, explorative thinking, and combining knowledge in novel ways. Sub-operations of this form of imagination include problem solving and expression, counterfactual reasoning, divergent thinking, hypothesis generation, and hypothetical reasoning (6, 50). Existing data suggests novel combinatorial imagination is also associated with activity in the default mode network. When tasked with the generation of novel open-ended ideas, the medial prefrontal and frontopolar cortex is activated, whereas more lateral aspects of these brain regions are associated with idea selection and determination of relevance (6, 51).

Phenomenology imagination reflects the engagement and response to esthetics that is largely subjective in nature. This form of imagination consists of attentional focus, cognitive engagement/appraisal, and emotional connection in relation to an object (52). Of the existing literature, esthetic appreciation was strongly associated with activity in the anterior insula irrespective of sensory modality (53), consistent with its role in interoceptive states of awareness (54, 55).

Lastly, Abraham (6) defined altered states imagination as those which involve a change in one’s awareness or mental state. This diverse range of altered states consist of dreaming, temporary induced states (e.g., use of psychedelics, hypnosis), or states associated with neurological and/or psychiatric symptoms (e.g., delusions, hallucinations, confabulation). Whereas dreaming is associated with activation of default mode network (56, 57) and deactivation of cognitive control networks (58), hypnotic states are associated increased activity in cognitive control networks (i.e., lateral prefrontal regions) and deactivation of the default mode network (59). This dissociation is thought to reflect the distinction between the involuntary vs. voluntary nature of dreaming and hypnosis, respectively. Meditation contains both volitional and nonvolitional elements and therefore includes activation of both the default mode and cognitive control networks (60, 61).

The capacity to imagine the future is a critical element of the human mind. While these processes and neuroanatomical correlates underlying the imagination have been discussed as discrete, the imagination has also been considered a “global emergent process” rather than something localized to a specific brain region (62). The radical imagination may be most consistent with the novel combinatorial form of imagination as it is geared toward the construction of ideas and things that have not yet happened. While there is evidence of the benefits of collectivism and working in community (63), including group wellbeing and support (63, 64), the field of neuroscience has yet to consider the brain health implications of what it means to imagine in connection with others who have a similar lived experience. Further, given the functional activation of sensory and motor cortices without direct sensory input (45), can the imagination initiate neuroplasticity and physiological changes that buffer cognitive decline? In addition to the political nature of the Black imagination, what cognitive health implications does collective dreaming have specifically for Black Americans, who are disproportionately impacted by Alzheimer’s disease and related dementias (ADRD) (19, 65)?

### 2.3. Imagination to promote neuroplasticity

Current evidence indicates Black Americans have approximately a 1.5 to 2 times higher risk of developing ADRD compared to non-Hispanic, white Americans (65–69). A significant proportion of this disparity is likely explained by the social and structural determinants of health (e.g., education, quality healthcare, community, and neighborhood built environment) with racial differences observed across many of these determinants due to historical and contemporary racism as well as unequal distributions of power (70).

For Black Americans, acknowledging the reality of present times while also imagining possible (freer) futures requires the difficult art holding of multiple truths: that racism is insidious and continues to disadvantage racial and ethnic minorities, but change is possible and necessary. Invocation of the radical imagination, through intentional shifts in one’s thinking about the construction of the future, may modify ADRD risk via neuroplasticity given the malleable nature of the brain even into late adulthood (71–73).

The concept of neuroplasticity has garnered interest in the field of dementia given the potential to influence cognitive and/or brain reserve (74) and delay clinical symptoms of cognitive decline despite underlying neuropathology. Neuroplasticity is defined as “the ability of the nervous system to change its activity in response to intrinsic or extrinsic stimuli by reorganizing its structure, functioning, or connections.” (75) Neuroplasticity can result in changes in neural structure, rewiring of existing neural connections, neurogenesis and synaptogenesis, or neurotransmitter and neurotrophin expression (76–78). Current data suggest external drivers of neuroplasticity are based on the novelty, complexity, and enriching nature of one’s environment (78). Thus, experiential exposure to education (79, 80), social engagement (81), and cognitively stimulating activities (82) are thought to support the brain’s capacity to resist the clinical manifestation of dementia (83, 84). Lifestyle factors, such as physical exercise and diet, also modulate neuroplasticity (83).

It is possible that one can modify the structural organization of the brain through the act of radically re-imagining an equitable society. Although lesser discussed, several internally-driven factors, related to the process of imagining, induce neuroplastic change. For instance, conceiving new ideas and integrating concepts that are seemingly unrelated requires cognitive flexibility, divergent thinking, and creativity. Routine engagement in these cognitive processes resulted in increased gray matter volume as well as functional changes in brain regions associated with higher level cognitive control (dorsal anterior cingulate cortex, dorsal lateral prefrontal cortex) and posterior brain regions among adults (85). Additionally, thinking about the future requires aspects of both episodic and semantic memory (86, 87) in that imagination requires, to some degree, an extraction of stored information and recombination of that information in new ways, which may also promote neuroplasticity (88). Visualization (i.e., the generation of mental imagery) is a powerful technique associated with neuroplastic change (89) and can be a simple way to tap into the imaginary and, quite literally, “see” ideas of the future. Taken collectively, there is evidence that neuroplasticity can occur in response to different forms of stimulation even if the stimulation is the result of mentally driven efforts as opposed to external ones. Thus, for Black Americans, designing the future and projecting oneself at its center is a revolutionary and necessary act that can also leverage the dynamic nature of the brain. The long-term brain health benefits of what it means to engage the radical imagination and build possible futures remains an area ample for study.

## 3. From imagination to radical hope

A vision for the future without belief that the outcome is attainable leaves little worth acting upon. Hope is the process of looking to the future with a desired outcome in mind and maintaining belief that the outcome can be actualized. Radical hope specifically adopts a social justice orientation (90) and is considered radical because the hope is driven by the desire for a future rooted in equity and questions of *when* equity will be achieved trumps questions of *how* it will happen. Radical hope is a core tenant of the framework for radical healing for People of Color and Indigenous Individuals (91), and emphasizes Black agency and the design of new futures through a decolonized imagination. Within this framework, radical hope is the fuel that keeps the vision ablaze.

Hope has been instrumental to the livelihood of Black Americans and is described as “a gift from [the] ancestors that fuels [our] will to survive racial trauma.” (92) Radical hope carried those kidnapped from the continent of Africa across the Atlantic Ocean to survive the atrocities of the Middle Passage and make it to the Americas. Radical hope is further evidenced in their pursuit of self-taught education and literacy while enslaved, which was key to physical and intellectual emancipation (93, 94) and subverted white domination. The belief that they could achieve freedom in times of precarity and horror exemplifies radical hope. It was the rebuilding of new cultural practices as the old ones were stripped – that is, the development of new traditions, rituals, and narratives that are now cultural staples in present-day Black America – that came from a space of radical hope and possibility (94).

If Black American history has taught us anything, it is that radical hope must not be extinguished or else run the risk of societal stagnation, or even worse, suppression. In author and poet Langston Hughes’ Harlem (A Dream Deferred) (95) published in 1951, he raises the important question:

*What happens to a dream deferred?*

*Does it dry up*

*like a raisin in the sun?*

*Or fester like a sore -*

*And then run?*

Without having to reference hope directly, Hughes’ lines of questioning challenges Black Americans to maintain the collective dream to upend the very real, harrowing happenings of that time: Jim Crow racial segregation, voter suppression, and police brutality. He advocates for Black Americans to have resolve–a steadfast hope–especially in times when the desired outcome is delayed or seemingly out of reach.

Black American hope has often been situated within a religious and/or political context and there are numerous examples in history of the symbiotic relationship between the Black Church and Black political action (96). We experience themes of hope in Martin Luther King, Jr’s infamous “I Have a Dream Speech.” At the Democratic National Convention in 1988, Reverend Jesse Jackson said, “Use hope and imagination as weapons of survival and progress, but you keep on dreaming, young America […] Keep hope alive!” (97) Hope is also laced throughout Barack Obama’s presidency and encapsulated by his campaign slogan, “Yes we can.” Radical hope is a throughline of the Black consciousness and imagination that keeps Black Americans afloat in times of precarity.

Most literature approaches the study of hope through a very Western, individualistic lens which emphasizes a bootstrap mentality and belief that the future is shaped exclusively by individual effort and self-determinism. It is important to distinguish this notion from that of radical hope which goes beyond individual desires and actions (98) – radical hope is about committing to a new collective future among a group of people experiencing injustice.

Although new frameworks are emerging that conceptualize hope as a culturally determined value [e.g., see Cherrington’s Afrocentric framework (99) and Mosley and colleagues’ psychology theory of radical hope (98)], most existing research on hope has come from a Western perspective including Herth’s psychological model (100) and Snyder’s Theory of Hope (101, 102). According to Synder, hopefulness is a cognitive process comprising three components: (1) *goals thinking* - clear conceptualization of a desired future outcome; (2) *pathways thinking* - the generation of routes and strategies to obtain the outcome; and (3) *agency thinking* - the perception that one can achieve those goals. This theory lends itself to examining potential neuroanatomical and functional correlates of hope.

### 3.1. Cognitive neuroscience of hope

Prefrontal brain regions likely underlie some components of hope given its emphasis on planning and positing future outcomes (103). Of the few studies examining the neural underpinnings of hope, one study found that higher dispositional hope was associated with lower fractional amplitude of low-frequency fluctuations (fALFF), a measure of fluctuation in resting state BOLD-fMRI signal, in the bilateral medial orbitofrontal cortex (104). Hope was also a significant mediator of the relationship between spontaneous brain activity in the orbitofrontal cortex and symptoms of anxiety. Several studies implicate the orbitofrontal cortex as a region responsible for reward processing and emotion-related learning (105, 106), such that human motivational states (i.e., willingness to act and engage in social behavior) is largely dependent on processing of rewards and punishers. This evaluative process may be closely linked to agency thinking (i.e., belief that one can achieve goals) in Synder’s model of hope given the orbitofrontal cortical influence on motivated behavior.

Another study found greater gray matter volume in the supplementary motor area (SMA) was associated with higher hope (107). The SMA, responsible for planning voluntary movements, is also important for mapping cognition to action. This includes inhibiting a response plan, alternating to a new response, and minimizing competing stimuli that interfere with task goals and cause distraction (108). Left SMA lesions are also linked to the executive control (mental manipulation) component of working memory (109) and most closely corresponds to pathways thinking hope.

There is a wealth of positive psychology literature that demonstrates the psychological and physical benefits of hope at the individual level [see (98) and (110) for an overview], which include greater life meaning and satisfaction (111–113), fewer symptoms of anxiety and depression (112, 114–116), less suicidality (112), and feelings of autonomy and purpose (117). Hopefulness is also associated with better recovery from physical illness and injury (116, 118, 119), and positive health behaviors (120).

A large body of work demonstrates that group self-efficacy (i.e., the shared belief that one’s group can achieve social change) predicts motivation to partake in collective action (121, 122). Feelings of hope are uniquely associated with Black Americans’ collective self-efficacy in that willingness to act on a social matter was present for Black Americans with high hope, but this association was not observed among white Americans in the sample (123, 124). This finding highlights the mobilizing power of hope for Black Americans and how hope is deeply woven into the fabric of Black American existence. Both hope and self-efficacy are necessary antecedents of action toward the future and speak to the unified reclamation of agency that is characteristic of the Black American culture. More studies are needed to understand what it means to hope within a community context and to have a shared sense of purpose.

In sum, radical hope “stretches the limits of what is possible…and belief that it is worth taking the next step” (96, 125). It requires Black Americans’ deep examination of our relationship with the past and the future, belief that an alternate future is possible, and a commitment to acting on that future (97, 126).

## 4. A communal courage

“I learned that courage was not the absence of fear but the triumph over it. The brave man is not he who does not feel afraid but he who conquers that fear.”

-Nelson Mandela.

The impetus behind any major social justice movement is imagining new ways of being and maintaining hope, but the spark that initiates action and fosters change is courage. Courage is the willingness to act upon imagined possibilities despite fear or possible failure. Asserting one’s presence in the future, in a day and age where Black lives are far too often ended prematurely, is a courageous and necessary act.

A core element of courage within a social justice context is that the motivation to act comes from the needs of the community. Most social justice movements have a figurehead who becomes the “face” of the movement, but the work is maintained and propelled forward via on-the-ground mobilization of the community. Thus, it is the collective action — the cooperative behavior of a group to achieve a common goal – that is the backbone of movements for equity. Existing literature suggests that collective action is driven by identity, perceived unfairness, and perceived efficacy (127). There is also alignment of emotions and values across the group which maintains a level of cohesiveness. Amongst the most critical of these shared values is courage. A society, or subgroup, can develop a collective emotional orientation, like communal courage, based on a shared sense of social identity (128). This develops as a byproduct of having shared cultural experiences, processes of socialization, and even racialization.

While there are many different conceptualizations of courage (129), Williams and colleagues (130) delineate the importance of *civil courage* – brave behavior that is specific to social change. This is differentiated from other forms of courage in that civil courage includes indignation about injustice (131). It is also distinguished by the fact that there is a major social cost, or even ostracism, at risk but action is taken regardless due to strong moral imperative (132). This form of courage is exemplified by the Little Rock Nine as the Black teenagers integrated the Arkansas high school in 1957 with escort from the National Guard after being harassed by anti-integrationists. Civil courage was also demonstrated by the four North Carolina A&T students who staged the 1960 Greensboro sit-ins and asked to be served food at a “white-only” Woolworth counter. In both circumstances (and many others), the Black American community moved in solidarity in demonstrating their unwavering support in response to these courageous actions. For example, the college students’ refusal to move and be served food in the Black standing area sparked a wave of 300+ students who joined the protest in solidarity and rippled to other regions around the country to initiate a sit-in movement. This courage in the face of injustice despite huge risk, including the risk of death, remains a testament to the Black American spirit.

Collective courage within a modern-day social justice context may be fostered through: (1) overlap in aspects of identity or lived experience or collective memory, (2) agreement and understanding of the root cause of injustice (e.g., racism), (3) identification of shared goals toward a greater purpose or belief, (4) tolerance of uncertainty and/or risk, and (5) belief that a just and equitable society is possible.

### 4.1. Cognitive neuroscience of courage

The decision to act in a prosocial manner is a conscious one that requires a specific level of evaluation about the consequences of acting. Civil courage specifically has been noted to rely on (1) internalization of social norms and (2) competency to act when needed (133). There is evidence that the ventrolateral prefrontal cortex plays a role in social reasoning and social norms (134). Additionally, civil courage requires adequate management of fear. Thus, courage is the pursuit of a desired outcome and the willingness to act despite the presence of fear. Fear is an evolutionary safeguard and primary emotion that arises in response to perceived threat or danger (128, 135). It is well established that the amygdala plays a primary role in processing threat and modulates the fear response (136–138). The ventral midline thalamus is additionally critical for one’s response to visual threat in that its nuclei, the xiphoid nucleus and nucleus reuniens, have projections to the basolateral amygdala and the medial prefrontal cortex, respectively (139). Activation of the latter pathway (nucleus reuniens ➔ medial prefrontal cortex) is responsible for the promotion of saliency and arousal. In animal models, activation of this pathway was associated with confrontational responses to threat (139) most consistent with the concept of courage.

One study with a human sample showed fMRI BOLD activity in the subgenual anterior cingulate cortex and right temporal pole was positively correlated with overcoming fear among volunteers who feared snakes but had a live snake moved toward them in an experimental paradigm (140). Somatic arousal (measured by skin conductance level) was attenuated as self-reported fear increased and the participant chose to overcome fear; somatic arousal was elevated in conditions where there was high reported fear and the participant choose to retreat/escape the threat. The subgenual anterior cingulate cortex may be part of the functional neuroanatomical network of courage in that it may inhibit fear-based somatic arousal and increase parasympathetic activity during courageous acts.

Courage operating at the collective level (i.e., communal courage) may have an influence on cognitive health and wellbeing via the social support afforded through ingroup membership, particularly when identifying with a group based on belief in a social justice issue. Social support promotes resilience to stress and adversity (141). Both stress and adversity are factors that have adverse effects on cognitive functioning via hypothalamic pituitary adrenal axis (HPA) dysregulation (142) and inflammation (143), and may increase risk for dementia (144, 145). Lifetime stress is associated with memory decline among middle aged Black Americans (146). In their systematic review of the literature, Kelly and colleagues (147) found higher levels of social support was associated with better general cognitive functioning, and less social support was associated with slower processing speed with a portion of these findings attributable to depression symptoms. Strong social support built through engaging in a collective goal or vision for the future may operate as a buffer against the stress that can come with acting in courageous ways.

## 5. Toward health and healing justice

The Black radical imagination is a conduit to possible futures rooted in health and healing justice for Black Americans. Both health and healing are liberatory for communities experiencing systemic oppression, which has been described as “society selectively concentrating trauma” (148). For those who are subjects of oppression, this trauma becomes embodied [i.e., integrated at the biological level (149)] and there is a wealth of evidence demonstrating its negative effects on Black bodies (16, 17, 20, 150) and minds (151–156). For example, the detrimental impact of oppression is evidenced in the links between systemic racism and Black women’s higher allostatic load (157) and advanced cellular and biological aging (158, 159). In fact, the weathering hypothesis (i.e., the postulation that Black women’s chronic exposure to stress to accelerates aging and results in health decline) was developed to characterize these environment-biological interactions specific to the intersectional oppression Black women experience (158, 160).

Both chronic stress and trauma contribute to changes at the neuroanatomical and cognitive levels (161–163). However, the neurological and neuropsychological impact of an American history fraught with racism is just now being more deeply explored. Racial discrimination, a direct form of race-based traumatic stress, is associated with lower total brain volume (164), lower fractional anisotropy in the corpus callosum, cingulum, and superior longitudinal fasciculus (165), and heightened activation in brain regions associated with threat vigilance (middle occipital cortex) and threat response (ventromedial prefrontal cortex) (166). There is also evidence that experiences of discrimination are associated with higher levels of spontaneous activity in the amygdala and stronger functional connectivity between amygdala with other neural regions (167) and future work is needed to determine whether this translates to an increase in physiological arousal and/or vigilance. These findings highlight how interpersonal encounters of race and identity-based mistreatment get “under the skin” to influence brain biology.

There is emerging evidence of the links between racism and ADRD among Black Americans (53, 70, 168). Cognitive aging is strongly impacted by the social environment and may be accelerated among Black American by exposures to structural racism across the life course. For instance, laws, public policies, and societal beliefs, shaped by structural racism, differentially allocate health-promoting resources and disadvantage racially and ethnically minoritized populations (169). Manifestations of structural racism can be observed in laws and practices impacting differences in educational quality (170), racial residential segregation (15), access to green space (171), political disempowerment/voter suppression (172), policing (152, 173), and mass incarceration (21, 22), all of which are associated with health inequity (174, 175) and may confer ADRD risk among Black Americans.

Collectively, these findings are confirmation of what Black people have long known – the body keeps the score. The Black body also tells its history. Trauma is transmitted across bodies and over time, and there is evidence that trauma manifests intergenerationally (176). As such, Black healing and health, down to changes initiated at the cellular level, are inextricably linked to Black liberation. Black healing is a liberatory praxis and can be achieved through the radical imagination.

Thus, part of the radical imagination encompasses Black people designing and discovering new ways of being in relation to one’s body to facilitate health and healing. This discovery must also center the experiences and desires of young Black girls (177) as well as elders -- those who exist at the margins of society and are overlooked in the process of knowledge production. Importantly, the onus should not be placed on Black people to “fix” a societal problem that was not designed by them; however, Black people deserve to and can live well despite existing injustices. Re-imaging collective wellness is one way of doing so.

The Kindred Southern Healing Justice Collective (178), a grassroots collective of southern healers and health practitioners who address trauma through models of collective wellness, is an excellent example of the radical imagination and healing in practice. Cara Page, one of the founding members of the collective introduced the framework of healing justice (179) – which “identifies how we can holistically respond to and intervene on generational trauma and violence, and to bring collective practices that can impact and transform the consequences of oppression on our bodies, hearts and minds.” Healing justice addresses significant gaps in Western medicine through its application of indigenous and ancestral knowledge systems and focus on holistic (mind, body, social, spirit) wellness. Healing within community for Black Americans may look like participation healing circles (180, 181), storytelling or oral tradition practices (182, 183), or engaging in rituals or ceremonies (184), which are culturally-affirming, strengths-based, and empirically supported approaches.

Little academic literature focuses specifically on healing justice as a framework, except for one systematic review (185), likely because “alternative” forms of healing are often overlooked due to epistemic exclusion (186, 187)--a general devaluating and delegitimizing of work that does not fit within Western ways of knowing or healing. However, a great deal of care has been given to healing justice in organizing and social justice spaces.

Extending the Black radical imagination to address issues of cognitive health disparities and the impact of systemic oppression on brain health is critical for Black aging futures and healing justice. Consider: What would it mean for Black Americans to have the space and time to dream of a better future and truly believe the desired outcome is possible? What would it look like for those outcomes to be actualized in this lifetime? What would Black healing look like at the deep cellular level, and what implications would it have for future incidence of ADRD and other physical and mental health conditions? It is through the radical imagination that we create new realities where all have an equal chance of making it to the future.

## Data availability statement

The original contributions presented in the study are included in the article/supplementary material, further inquiries can be directed to the corresponding author.

## Author contributions

The author confirms being the sole contributor of this work and has approved it for publication.
